# Reports on the Hospitals of the United Kingdom

**Published:** 1910-03-26

**Authors:** Henry Burdett


### REPORTS
### ON THE
### Hospitals of the United Kingdom.

By SIR HENRY BURDETT, K.C.B., K.C.V.O.

### XXXIV.—HUDDERSFIELD INFIRMARY.

This institution was established in 1831. It occupies a conspicuous site, being erected in the Public Gardens and united with them. There are Turkish and medicated baths, which are also open to the public. The grounds are very tastefully laid out, and although the atmosphere is against floriculture, still the results are very picturesque and on the whole satisfactory. The infirmary contains 140 beds, with an average occupancy of 100. The general excellence of the management, in which leading citizens take a genuine interest, is proved by the fact that when we inspected it in February 1893 we were able to testify to the careful administration and close attention to detail which were everywhere apparent. That opinion we are able to confirm by our inspection on October 16, 1909, when we were struck by the splendid smartness which is maintained throughout the hospital, with the exception of the operating theatre. It is not too much to say that the neatness and order which are everywhere present throughout the entire institution are remarkable and praiseworthy.

The Board-room contains some excellent portraits of departed worthies, some of them rendered historical by the ancient custom whereby donors of £2,000 seem to have had their portraits painted and hung in the Board-room.

### The General Condition.

The general condition of this hospital is good; everything is beautifully kept. The wards, containing 32 beds, were airy and comfortable, owing to the fact that they are very wide and spacious, and that it is possible to flush them with air by opening the doors leading on to the balconies. The wards in the new wing, where there is a system of artificial ventilation, were not adequately ventilated, and presented rather a striking contrast in this respect to the older wards. The top floor of this wing was occupied by children, and a close study of the condition of the children as we found them convinced us that every child in these wards was air-starved and was suffering in consequence. The experience of St. Thomas's Hospital proves that in the English climate artificial ventilation is not desirable, especially in children's wards, and that those who permit this system to continue incur a very heavy responsibility, for the cases in wards so ventilated do not progress as rapidly, if they even progress at all, as they ought to do under modern treatment in a large aseptic ward, when an abundance of outside air is constantly passing through the whole of its extent.

The Mayor of Huddersfield has taken an active and worthy part in the life of the city by making himself the champion of the rights of little children. The committee of this hospital have always shown an earnest desire to maintain its institution at the highest state of efficiency, and we have, therefore, every reason to hope that prompt steps will be taken to give up the artificial system of ventilation for the children's wards, and to substitute for it natural ventilation, with open windows and an abundance of fresh air. Until this has been done, in our view the fewer children that are admitted to this hospital the better.

### An Example with a Moral.

Dr. Bristowe and Mr. Timothy Holmes, in their report to the Privy Council on the hospitals of the United Kingdom in 1863, made a remarkably apposite statement concerning the York County Hospital when they visited it. They declared that the York Hospital is a well-constructed hospital like that at Huddersfield, that it is on open ground with ample accommodation for the patients, that the administration is good, and much more, but that the introduction of an artificial system of ventilation so affected the patients that the surgeons had refused to operate. Remembering this, we brought the matter at once to the notice of the Mayor of Huddersfield, who has taken an active and worthy part in the life of the city by making himself the champion of the rights of little children. This was in October 1909, and we have much pleasure in being able to state on his authority that in consequence the children's ward at the Huddersfield Infirmary, and the ventilation and heating system attached to it have been entirely altered, so that the children and other patients in this wing have now the advantage of a plentiful supply of fresh air. The altered system has been tested for a few weeks, and the medical staff, with the nurses, agree that the ward is now satisfactory, and it has been determined to apply the altered system of ventilation and heating to the other wards where such a course was indicated by the conditions to which we have just called attention.

### Some Practical Considerations.

We noticed in the wards an excellent trolley, which must prove very useful in dealing with patients when they are first able to leave their beds for a short time. These trolleys are picturesque and attractive, and we should like to see at least one of them in the large ward of every hospital.

Another rather curious and unique feature of the furniture of the wards is the pattern of patients' lockers. These are large and are surmounted in some wards by a cross, and in other wards by a circle. What precise meaning is attached to either symbol we were unable to ascertain, though there is evidently some motive or principle behind the design which the staff might usefully fathom and reveal to the world.

Another matter which is worthy of record is the experience in this hospital, and also in the case of the Halifax Infirmary and other institutions, that the quality of the porcelain of the baths and slop sinks, supplied by well-known firms, is inferior and not suitable for hospital purposes. It is false economy not to pay an adequate sum for baths, slop sinks, and other appliances of the kind, and no contract should be accepted by any hospital for wares of this description unless it contains the condition on which Messrs. Doulton accepted their contract—that is, that the supplying firm will replace, free of cost, every bath and appliance of the kind which cracks or shows signs of defective wear within a period of three years from the time it is fixed. It is but just that we should record that throughout our inspections we have never seen any porcelain baths, slop sinks, or similar appliances made by Messrs. Doulton which have exhibited the defects which are so lamentably apparent in the wares supplied by other firms. That a porcelain bath can be made for hospital purposes which will endure without defect for 20 years and upwards is proved by the fact that Rufford baths are to be found at Huddersfield and in many other hospitals which, though they have been in use for this long period of years, are in an effective and useable condition to-day. Architects who have attained a high position as constructors of hospitals in this country are sometimes too proud to schedule second-class goods, and in one or two of the newest hospitals these goods so supplied are already exhibiting defects which prove them to be unsuitable for hospital purposes.

### Mortuary and Post-mortem Unit.

The mortuary is efficient, though it represents an old type of construction now out of date. The condition of the post-mortem room was such as to make it a marked feature in this well-kept hospital, for it was the only place which was really dirty and not a credit to the institution. It presented the appearance of being little used and altogether neglected. We hold that the mortuary and every part of its adjacents should be maintained in a high state of cleanliness, for there the hospital is able to set an example to the whole community of reverence for the dead by exhibiting respect to the living, whether the latter be friends of the deceased or officers of the hospital.

### A Few Other Matters.

Finally, we would advise that the circular metal boxes, which are of a very old and defective pattern, used in the operation theatre should be abolished. They are not aseptic. Having regard to much that is excellent in the theatre, it is but just that more up-to-date appliances should be used. We think, too, that the instruments require to be more frequently overhauled, and that many of them should be kept in better condition than they are at present.

We are surprised to see that in a business community like that of this hospital the accounts of the institution are not kept on the uniform system. It is not possible, therefore, at the present time to compare the working of this hospital with that of others of the same size. This seems to us a great injustice to the Huddersfield Infirmary, where economy and good management seem to work hand in hand, to the great advantage of everybody concerned. The adoption of the uniform system of accounts would put this matter right. We shall hope to hear of the treasurer giving instructions for the system of book-keeping to be altered accordingly, with the object of enabling full justice to be done to this hospital and its work.

We are glad to note that the hospital appears to be well supported both by the workpeople and also by other members of the community. The hospital has invested funds exceeding £60,000; the expenditure is under £9,000 a year, so that the institution has a good balance of capital in hand to meet future emergencies.

---

The **Bridgewater Hospital,** the **Leatherhead (Victoria Memorial) Cottage Hospital,** and the **Wells General Hospital** will be reported on next week.

